# Distal nerve transfer (PT-AIN, SUP-PIN) for regaining hand function in C8, T1 root injury following extirpation of the right C8, T1 schwannoma

**DOI:** 10.3171/2022.10.FOCVID22110

**Published:** 2023-01-01

**Authors:** Lukas Rasulić, Andrija Savić, Jovan Grujić, Aleksa Mićić, Stefan Radojević, Jasmina Ivić, Milan Lepić

**Affiliations:** 1 Faculty of Medicine, University of Belgrade;; 2 Department of Peripheral Nerve Surgery, Functional Neurosurgery and Pain Management Surgery, Clinic for Neurosurgery, University Clinical Center of Serbia, Belgrade;; 3 Clinic for Plastic Surgery and Burns, Military Medical Academy, Belgrade; and; 4 Clinic for Neurosurgery, Military Medical Academy, Belgrade, Serbia

**Keywords:** C8, T1 root injury, distal nerve transfer, regaining hand function

## Abstract

A 48-year-old female was admitted to the authors’ department due to hand weakness as a consequence of C8, T1 root injury. Eight months earlier, the patient had been treated by a pulmonary surgeon due to an expansive lesion near the apex of the right lung, which resulted in right lower brachial plexus palsy. Postoperative pathohistological findings indicated that the lesion was nerve schwannoma. The diagnostic process included physical examination, electromyoneurography, and MRI. A distal nerve transfer (pronator teres–anterior interosseus nerve [PT-AIN], supinator–posterior interosseus nerve [SUP-PIN]) was performed in order to restore hand function.

The video can be found here: https://stream.cadmore.media/r10.3171/2022.10.FOCVID22110

## Transcript

Distal nerve transfer (pronator teres–anterior interosseus nerve, supinator–posterior interosseus nerve)^1,2^ for regaining hand function in C8, T1 root injury following extirpation of the right C8, T1 schwannoma.

### 0:35 Indications for Distal Nerve Transfer.

The indications for distal nerve transfers are following: upper brachial plexus palsy (C5–6), lower brachial plexus palsy (C8–T1), isolated axillary nerve lesion, isolated proximal extensive radial nerve lesion, isolated musculocutaneous nerve lesion, isolated proximal extensive median nerve lesion, isolated proximal ulnar nerve lesion.^3^

### 1:02 Patient History.

The 48-year-old female was admitted to our department due to hand weakness as a consequence of C8, T1 root injury. Eight months earlier, the patient was treated by a pulmonary surgeon due to expansive lesion near apex of the right lung, which resulted in right lower brachial plexus palsy. Postoperative pathohistological findings indicated that the lesion was nerve schwannoma. At the admission, clinical findings included right lower brachial plexus palsy and pain in the C8 and T1 dermatomes of the right thorax and arm. Electromyoneurography (EMNG) revealed acute axonal lesion in the C8 and T1 myotomes of the right arm. Magnetic resonance imaging (MRI) showed a lesion of the C8 and T1 roots at the level of the right transversal process of T1 vertebra.

### 1:54 Patient Positioning.

The patient was placed in the supine position.

### 1:59 Demonstration of Initial Incision.

The S-shaped incision was made at the volar side of the forearm.

### 2:07 Exploration Onset.

The nerve exploration was started by dissection of soft tissue layer by layer.

The antebrachial fascia is dissected.

### 2:50 Identification of the Deep Radial Nerve Branch.

The deep radial nerve branch was identified and marked with the red catheter. The anatomical landmarks for deep radial nerve branch exploration are noted: 1) the brachioradial muscle, 2) the lateral compartment extensors (extensor carpi radialis brevis and longus), and 3) the supinator muscle and the arcade of Frohse.

The fascia from the biceps distal insertion is dissected.

### 3:52 Identification of the Median Nerve Branch.

 The median nerve was identified and marked with the blue catheter. The anatomical landmarks for the median nerve exploration are noted, the arcade of the flexors and the pronator teres muscle.

### 4:06 Identification of Terminal and Side Branches.

Further exploration included identification of terminal and side branches: 1) branch for pronator teres, 2) anterior interosseus nerve, 3) superficial sensory radial nerve, 4) posterior interosseus nerve, 5) branches for the supinator.

### 4:32 Marking of the Nerves and Branches of Interest.

The nerves and the branches of interest for this procedure in this individual patient are marked.^4–8^

### 4:40 Direct Nerve Stimulation.

By direct stimulation, the donor branches (for supinator and pronator teres muscles) and recipient nerve branches (PIN and AIN) were verified.

### 4:55 Carrying Out the Transfer.

Preparation of the first recipient branch, the posterior interosseous nerve. The application of bands infused with an adrenaline solution (1:10) for preventing significant bleeding at the nerve stump. Preparation of the branches for supinator muscle in order to provide a first donor branch. The proper positioning of the recipient and donor branches is necessary, so there is no tension over suture line. The epineural suture provides positioning of the branches with minimal damage to the tissue. Combination of 1 to 2 sutures with 1 ml of fibrin glue provides stabilization of the suture line without tension. The same process was repeated to make the second transfer. Following the nerve transfer, the adrenaline infused bands were carefully removed.

### 10:07 Closure Technique.

The layered closure technique included cutaneous and subcutaneous sutures of the dissected tissue.

## Disclosures

The authors report no conflict of interest concerning the materials or methods used in this study or the findings specified in this publication.

## Author Contributions

Primary surgeon: Rasulić. Assistant surgeon: Savić, Grujić. Editing and drafting the video and abstract: Grujić, Mićić, Radojević, Ivić, Lepić. Critically revising the work: Rasulić, Grujić. Reviewed submitted version of the work: Grujić, Lepić. Approved the final version of the work on behalf of all authors: Rasulić. Supervision: Rasulić.

## Supplemental Information

### Patient Informed Consent

The necessary patient informed consent was obtained in this study.
